# Estimation of infant vaccination coverage at national and region level in Ethiopia: a cross-sectional study

**DOI:** 10.11604/pamj.2022.42.101.18299

**Published:** 2022-06-07

**Authors:** Aschalew Teka Bekele, Mulat Nigus, Liya Wondwossen, Thomas Karengera, Ephrem Tekle, Pamela Mitula, Lemlem Assefu, Teklay Kidane, Woldemichael Yonas

**Affiliations:** 1 Immunization Officer World Health Organization, Ethiopia Country Office, Addis Ababa, Ethiopia,; 2 Federal Ministry of Health, Addis Ababa, Ethiopia,; 3 World Health Organization, Ethiopia Country Office, Addis Ababa, Ethiopia

**Keywords:** Immunization coverage, Ethiopia, WHO-UNICEF estimate

## Abstract

**Introduction:**

an increasing trend of routine immunization performance has generally been observed over the past decade in Ethiopia. However, inconsistencies were observed over time and among different sources of data. This review analyzed systematically data from various sources and produced regional and national coverage estimates for antigens offered in the infant immunization program in Ethiopia.

**Methods:**

we collated data from administrative reports, population-based surveys and other sources to produce annual estimates of vaccination coverage. We obtained relevant data for each of the 9 Regional States and 2 city administrations, for the period 2007-2016. Region level estimates were produced based on survey results, interpolation between or extrapolation. We aggregated the resulting region level estimates, using a population-weighted approach, to give national estimates.

**Results:**

we found that the national Penta 3 coverage of Ethiopia increased from 59% in 2007 to 71% in 2016. For the 110 vaccination estimates produced at region level, 71 were based on interpolation or extrapolation from empirical anchor points; 18% were based on surveys and 17% were based on administrative data.

**Conclusion:**

while we recognize the critical importance of improving the quality of information on vaccination coverage from administrative reporting systems, we are also cognizant of the expected continued need for region level surveys and improved rapid-monitoring exercises.

## Introduction

The immunization program in the Federal Democratic Republic of Ethiopia was launched in 1980. The program currently provides antigens against 11 vaccine preventable diseases aiming to protect more than 3 million births annually. The immunization program has contributed to the reduction of child mortality through the provision of life saving vaccines to increasingly a greater number of children [1].

Under the 2014-2015 governmental plan for improving routine immunization services, technical assistants were deployed in 51 priority zones and health posts were equipped with solar powered refrigerators. The 2015/16-2019/20 Health Sector Transformation Plan (HSTP) calls for providing quality and equitable health services to all citizens [2].

In 2009 a nationally integrated health management information system was launched whereby immunization related indicators are reported from health facilities to the national level. Information on vaccination coverage is also collected from different sources including coverage evaluation surveys, service availability and readiness assessments, nationally representative data quality self-assessments, surveillance on vaccine-preventable diseases and vaccine stock monitoring reports. However, the monitoring of trends in vaccine coverage is complicated by the multiple sources of relevant data and the varied quality of those sources [3]. In Ethiopia, as elsewhere, the accuracy of estimates of coverage based on administrative reports depends on the accurate recording of the numbers of administered doses; accurate information on the size of the target population; regular and robust reporting by the health workers who administer the vaccines; and the prompt and accurate transfer of the relevant data through all of the levels between health posts and the national government. The denominators needed to calculate percentage coverage values are based on the 2007 census which provides only rough estimates.

As nationally representative surveys of vaccination coverage are expensive and time-consuming, they tend to be infrequent and inadequate to provide rapid information on the trends in a system´s or programme´s performance [4]. In Ethiopia, the most recent EPI coverage survey was conducted in 2012 while the most recent Demographic and Health Survey was conducted in 2016 [5]. Often administrative coverage data have served as the primary vehicle for the annual monitoring of vaccination coverage and programme performance at national and sub-national levels. To improve the timely availability of data the Government initiated the implementation of an electronic Health Management Information System (eHMIS), established performance review teams in health centres and conducts periodic national data quality assessments. However, the reliability of a single information source alone is challenged when inconsistencies are observed between coverage results from the administrative monitoring and coverage surveys [6].

In an attempt to improve our knowledge of recent trends in child vaccination coverage in Ethiopia, we recently collected relevant data from multiple information sources and used them to derive estimates of the annual levels of such coverage, at both region and national level, for the years 2007-2016.

## Methods

**Study design:** this was a cross-sectional study carried out from 5-8 September 2017 in Addis Ababa, Ethiopia. The study analyzed 10 years of administrative and population-based coverage survey data and applied inferential rules to produce coverage estimates.

**Setting:** a national data quality review workshop was organized in Addis Ababa Ethiopia to analyze available immunization coverage data using various sources of information. The national review team organized a four-day data quality review workshop from 5-8 September 2017. The meeting venue was a private meeting facility in Addis Ababa. During the workshop representatives from each region presented their data and clarified any apparent discrepancies observed such as wide year to year variations.

**Participants:** participants were drawn from the national level, and region levels and vaccinators from two districts. Regions were represented by their immunization and HMIS focal persons; The national review team members included experts from i) Central Statistics Office (CSO), ii) national immunization program and planning policy directorate of the Ministry of health; iii) immunization and data experts from country offices of the World Health Organization (WHO) and United Nations Children´s Fund (UNICEF) and iv) the WHO Intercountry Support Teams for East and Southern Africa (WHO IST/ESA) and WHO Headquarters.

**Data sources/measurements:** the review team obtained region-specific vaccination coverage data from administrative reports and population-based immunization and health surveys.

**Variables:** we investigated data on BCG, the first dose and third of diphtheria, tetanus, pertussis, hepatitis B and haemophilus influenza type b vaccine (Penta 1, Penta 3), the third dose of oral polio vaccine (OPV3), the first dose of measles vaccine (MCV1), the third dose of pneumococcal conjugate vaccine (PCV3) and the second dose of rotavirus vaccine (ROTA2).

In Ethiopia, the planning and budgeting cycle runs from 8 July to 7 July of the following year. However, the country produces annual reports using the WHO/UNICEF Joint Reporting Format on Immunization covering the period January-December for each year. The review on PCV and Rota was limited to the period 2012-2016 and 2014-2016 as PCV and Rota vaccines were introduced respectively, in October 2011 and November 2013.

**Data processing and analysis plan:** the data reported through the routine health management information system (HMIS) covering 10 annual birth cohorts as well as the 2011 and 2016 Demographic and Health Survey (DHS) were investigated. Data were organized by region, vaccine and year, in an excel database. Temporal trends in coverage in each year and region were displayed graphically, with a different symbol used for each type of source of information.

For the data review and estimation process, we followed the domain-specific and logical inference rules previously used by WHO and UNICEF to estimate coverage levels in 195 countries [7,8]. For example, if there were no other relevant data available - or, at least, no other data that indicated a coverage value that differed by more than 10 percentage points from the reported administrative coverage - we took a reported administrative coverage as our estimate of the actual coverage. We also adopted the principles that reported state-level coverage could not exceed 100% and that any observations of large year-to-year decreases or increases in coverage are unlikely to be accurate unless there is some reasonable explanation - e.g. a vaccine stock-out or a strike by health workers. If no such explanation was apparent, we assumed that any data suggesting a year-to-year change in coverage of more than 10% were inaccurate and ignored them.

When, for a given year, region and vaccine coverage data were available from at least two different sources - e.g. from a report of administrative coverage and from a household survey that year was categorized as a so-called anchor point. Because the 2011 DHS and the 2012 EPI coverage survey were done within short intervals, the review team agreed to use the EPI coverage survey as the sample size was larger and included health facility visits to verify vaccination status from registers in the case when parents did not provide reliable information on the vaccination status of children. For most of the study Regions, the first anchor point was established in 2011 - Coinciding with the 2012 National EPI coverage survey and a second anchor point was in 2016-coinciding with the 4^th^ Ethiopian Demographic Health Survey. If the data from a survey did not support the corresponding reported administrative coverage - i.e. if it did not indicate a coverage that was within 10 percentage points of the reported administrative coverage-our estimate for that year was based on the survey results; otherwise, our estimate from that year was based on coverage estimated from administrative reports.

Once anchor points were identified and an estimate was assigned for those years estimates between anchor points were made on the following conditions: for years between two anchor points, in which surveys supported administrative data, our estimates were based on administrative reports. If administrative data were missing for any of those years an estimate was made based on simple interpolation between reported coverage. For years between two anchor points, in which survey results did not support administrative reports for at least one anchor point, our estimates were based on coverage trends established by administrative results calibrated to levels of the survey coverage.

For estimate beyond either the most recent or earliest anchor point if the anchor point value was based on administrative data the estimate was assigned the value of the administrative results for the remaining years. If the anchor point value was based on survey results the estimates were based on the administrative data calibrated to the level of the anchor point value. After producing a time series for each state-vaccine combination, we compared estimates across vaccines to ensure internal consistency and tried to resolve any apparent anomalies - e.g. substantial differences in the coverage for DTP3 and the third dose of oral polio vaccine. Whenever data from two or more information sources appeared to conflict, we tried to identify the most accurate source by consideration of the possible biases. All decisions underlying each estimate were documented. We aggregated region level estimates to produce National estimates. Using estimated annual birth projections from the Central Statistics Office, we computed region-specific weights for each study year by dividing the annual estimated number of births in each state by the corresponding estimated total number of births in Ethiopia. The weighted national mean coverage for each vaccine and year was then computed by multiplying each region level coverage by a region-specific population weight and then summing across the 9 Regional States and 2 city administrations.

## Results

**Figure 1:**
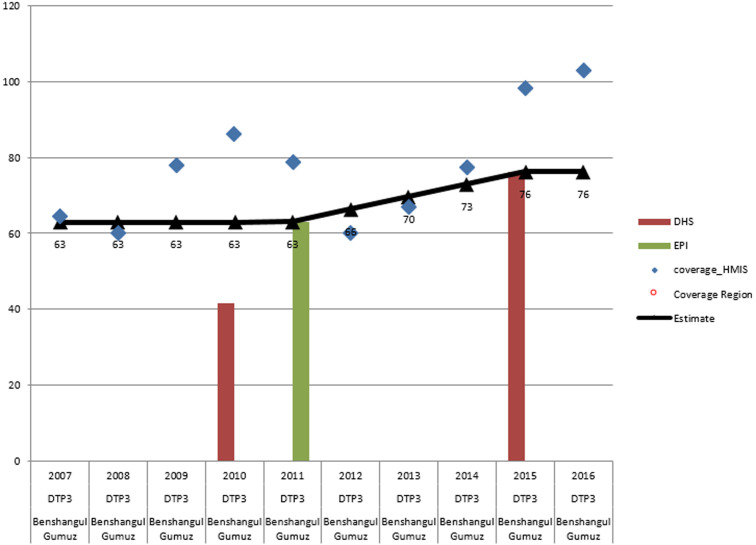
Penta 3 coverage estimate for Benishangul Gumuz Region, 2007-2016, Ethiopia

We produced estimate for each region and administrative city for the panel of vaccines recommended in the national immunization program. Estimate for each year and region are available from the corresponding author. Below we present an example of our estimate for Penta 3 vaccination in Benishangul Region (Figure 1). In 2011, which corresponded with the first anchor point, the estimate for this region and the selected vaccine was made by investigating three data sources: DHS 2011, the 2012 EPI coverage survey and DHS 2016. The DHS 2011 report provided coverage estimates for the 2010 birth cohorts while the EPI coverage survey and the DHS 2016 reports showed coverage estimates for the birth cohorts in 2011 and 2015 respectively. The 2011 DHS coverage survey report was ignored for all regions because the sample size was not appropriate for regional estimation. The EPI coverage survey was done in 2012 with regionally representative sampling and with efforts made to verify vaccination history by visiting health facilities. The first anchor point was established in 2011 based on the result of the 2012 EPI coverage survey which showed 14% difference from the reported data. The second anchor point was established in 2015 based on the DHS 2016 result which employed a similar methodology as the 2012 EPI coverage survey. The reported data from 2007-2010 showed year to year fluctuation without plausible explanation, therefore we estimated the coverage based on the nearest anchor point which is the value of the coverage survey in 2011. For the years 2011-2015, the coverage was estimated based interpolation between the 2012 and 2016 coverage survey results and the administrative report was ignored because of significant unexplained fluctuations in reported coverage.

**Figure 2:**
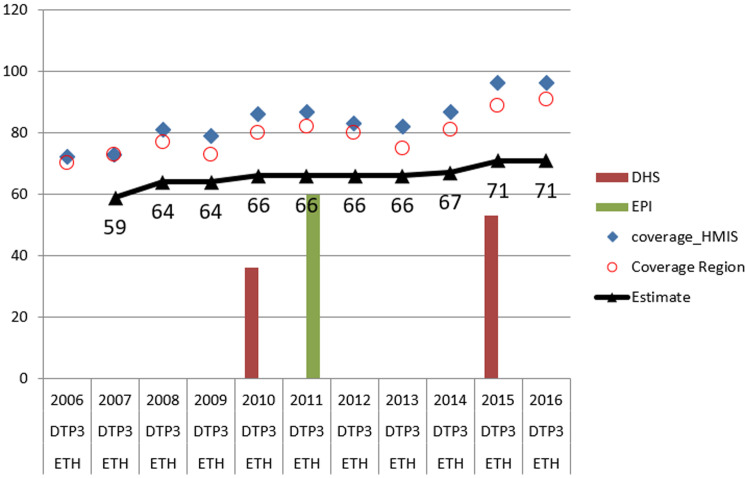
comparison of national coverage estimates for Penta 3 with different sources

**Figure 3:**
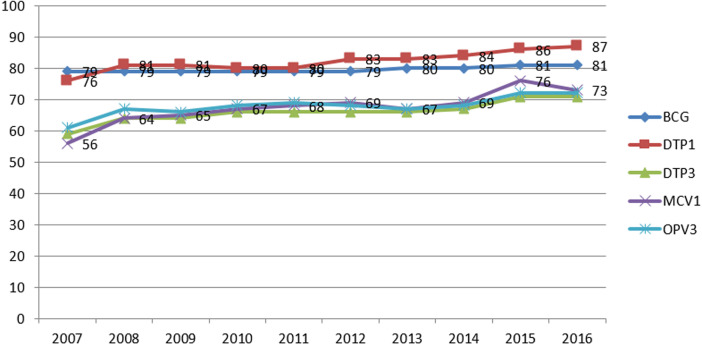
estimate of national coverage with five routine child vaccinations, 2007-2016, Ethiopia

Our estimates of coverage were often only based on interpolation or extrapolation of survey results, which frequently challenged the corresponding reported administrative coverages. For example, of the 110 estimates made for Penta 3, 71 (65%) were based either interpolation or extrapolation from the 2011 or 2015 anchor points. Moreover, 20 (18%) were based solely on survey results and the remaining 19 (17%) on reported administrative data. Similar patterns were observed for the other vaccinations we investigated. Where available, the results of nationally representative surveys were found to be generally supportive of our estimates of national coverages, which we derived from the region level values (Figure 2). Across vaccines, our estimates of national coverages tended to be lower than the corresponding reported administrative coverages (Figure 3). Between 2007 and 2014, estimated national coverages tended to gradually increase: from 79% to 81% for bacille Calmette-Guérin vaccine; from 76% to 87% and from 59% to 71% for Penta 1 and Penta 3, respectively; from 61% to 72% for the third dose of oral polio vaccine; and from 56% to 73% for the first dose of vaccine against measles. Coverages seem to have plateaued between 2013 and 2016. Over the same period, there appeared to be substantial reductions in drop-out between Penta 1 and Penta 3.

## Discussion

The results reported here reflect efforts by the Government of Ethiopia to review the available data on region level vaccination coverage systematically and produce better region level and national estimates of vaccination coverage. They also reflect a growing awareness of the challenges of estimating actual vaccination coverages from reported administrative coverages when their quality is challenged by other sources such as surveys. The government employed several initiatives to correct the deficiencies in the coverage monitoring system. A revised strategy for the monitoring of routine immunizations through the HMIS was launched in 2009. This was later transformed into electronic coverage monitoring system and community-based health information system whereby families in each household are registered and record kept at health post level. While we recognize the critical importance of improving the quality of information on vaccination coverage from administrative reporting systems, we are also cognizant of the expected continued need for region level surveys and improved rapid-monitoring exercises. Although the method of our data review may have fallen short of optimal, we found both the review and subsequent estimation exercise to be useful. The approach we followed allowed the evaluation and synthesis of multiple sources of coverage data, permitted some judgment of data quality and promoted a commitment to improved documentation.

Figure 1 illustrates the considerable differences we observed between the reported administrative coverages and our corresponding estimates of actual coverage. Variations were observed among regions in the maturity of the health information system where Tigray, Addis Ababa and Dire dawa regions showed to have consistent reporting system. Moreover, since recent years, the monitoring system in Amhara, Oromia and SNNPR tended to mature while it is relatively not mature in the other regions. Coverage for newly administered vaccines has shown inconsistencies in majority of the regions which needs to be addressed in the future planned introductions. Our approach to the estimation of coverages included the use of existing data, the assessment of all available Region-vaccine-year combinations, the inclusion of diverse data sources, the application of a set of logical inference rules to the data review, use of input from stakeholders and the flexibility to override the inference rules - with justification and documentation of the decisions taken. This approach is only as good as the available data. Missing data and data of poor quality restrict attempts to produce accurate estimates. Our estimates of coverage remain subject to error and may well be inaccurate even when they appear to be well supported by data from multiple sources. The annual collection of data for the estimation of national vaccination coverages may well be critical to evaluating progress in the elimination of vaccine-preventable diseases. In Ethiopia, although ever more children receive the benefits of vaccination, many children remain unvaccinated. Our estimates indicate that 71-73% of the Ethiopian 2015 birth cohort received Penta 3, third doses of oral polio vaccine and first doses of vaccine against measles. Although these results indicate that there have been substantial increases in coverage since 2007, much work will still be needed to reach the HSTP goals. At the same time, efforts need to be made to ensure that appropriate commitment and investment are being made and that coordinated and coherent action is being taken to improve Ethiopia´s programmes of routine immunization. In addition, Regular systematic data review should be implemented at the different levels of the health system to improve the quality of immunization data.

## Conclusion

In conclusion, the routine delivery of childhood vaccination coverage in Ethiopia has shown improvement over the period from 2007-2016. We observed inconsistencies between the administrative reported and survey data in most of the regions. We recommend that administrative recording and reporting of childhood vaccination needs to be improved and survey methods standardized for comparability. We also recommend systematic data review to be conducted regularly at the different levels of the health system.

### What is known about this topic

- Annually WHO and UNICEF publish national immunization coverage estimates for countries.

### What this study adds

- The study added one level of data analysis by using regional estimates to produce national estimates;
- The study increased capacity of country level staffs on conducting country level estimates by using various information sources;
- Because the study was led by country team, this facilitated acceptance of the new estimate despite its variation from the administrative coverage report.
